# What is the impact of rural-to-urban migration on exclusive breastfeeding: a population-based cross-sectional study

**DOI:** 10.1186/s13006-020-00330-8

**Published:** 2020-10-14

**Authors:** Xiao Han Yin, Chen Zhao, Yu Mei Yang, Hui Feng Shi, Tian Chen Wu, Jia Lei Xie, Jie Qiong Niu, Xiao Li Wang, Jin Fang

**Affiliations:** 1 Department of Maternal and Child Health, School of Public Health, Peking University, Beijing, China; 2 China Development Research Foundation, Beijing, China; 3 School of Economics and Management, Beijing Forestry University; China Center for the Economics of Human Development, Beijing, China; 4 National Health Commission Key Laboratory of Reproductive Health, Beijing, China

**Keywords:** Breastfeeding practice, Prevalence, Association, Rural-to-urban migration, China

## Abstract

**Background:**

In China, less than one third of infants under 6 months of age are being exclusively breastfed. Maternal rural-to-urban migration contributes to these low rates of breastfeeding practices. Therefore, the aim of this study was to assess the prevalence of breastfeeding practices and associated factors among rural-to-urban migrant children and local children with infants aged 0–12 months in China, 2018.

**Methods:**

Data were collected from a population-based cross-sectional survey in 2018 that included 6995 infants from eight urban areas (four metropolis and four medium sized/small cities) in China. The prevalence of breastfeeding practices was calculated using a 24-h recall questionnaire for all infants aged under 12 months. Logistic regression was conducted to examine the association between the prevalence of breastfeeding practices and maternal migrant status, after adjusting for sociodemographic characteristics, mother-infant health information and supportive information. For exclusive breastfeeding, we further analyzed its association with maternal rural-to-urban migration, stratified by maternal education level, maternal resident place and maternal ethnicity, respectively.

**Results:**

The overall prevalence of ever breastfeeding, exclusive breastfeeding, predominant breastfeeding and age-appropriate breastfeeding (exclusive breastfeeding of infants under 6 months of age and complementary feeding from six to 12 months of age) was 97.51, 29.84, 59.89 and 45.07%, respectively. Rural-to-urban migrant children were less likely to be exclusively breastfed compared to local children (AOR 0.81, 95% CI 0.68, 0.95). Stratified by different sociodemographic variables, a negative association between exclusive breastfeeding and rural-to-urban migration was only found in the group with high education level, in the group living in metropolis and in the group of minorities, respectively.

**Conclusions:**

The overall prevalence of breastfeeding practices was low in both rural-to-urban migrant children and local children. Besides common strategies, special approaches should be provided for urban highly educated migrants.

## Background

Considering the benefits of breastfeeding for both infants and mothers, the World Health Organization (WHO) recommends exclusive breastfeeding (EBF) for the first 6 months of life and introduction of nutritionally-adequate and safe complementary (solid) foods at 6 months together with continued breastfeeding up to 2 years of age or beyond [1]. However, inequity has been found between different countries, as well as within certain countries [2]. As a member of WHO, China launched the National Nutrition Plan (2017–2030), aiming to increase the EBF rate to at least 50% by 2020. Nevertheless, several studies conducted nationwide demonstrated suboptimal outcomes [3, 4]. The weighted prevalence of ever breastfeeding, exclusive breastfeeding among all infants under 6 months of age, breastfeeding at 1 year of age and breastfeeding at 2 years of age were 79.6, 20.8, 11.5 and 6.9%, respectively in 2013 [3].

Breastfeeding practices are predicted by demographic factors (i.e., maternal age, education, employment, income, residence), health-related factors (like parity), social support and policies [5]. However, few studies have examined the potential influence of rural-to-urban migration on breastfeeding practices. According to some studies, rural-to-urban migrant population are more vulnerable to health-related issues such as unhealthy lifestyle [6], low rate of medical treatment seeking behaviors [7, 8] and deficient disease management [9]. An increase of rural-to-urban migrant population in China has been noticed since 2000 and it is expected to reach nearly 300 million before 2020 [10, 11]. Many infants and children have also moved into cities with their parents for better health-related services and education.

Studies about the association between breastfeeding practices and residence inconsistency mainly focused on migrants who are living or giving birth in foreign countries [12–16]. These studies all concluded that the prevalence of breastfeeding practices was different between migrants and local people. To our knowledge, there are few studies focusing on breastfeeding practices among rural-to-urban migrant children in China. Considering the sensitivity of rural-to-urban migrants to health issues, it is reasonable to assume that there are differences in breastfeeding practices between rural-to-urban migrant children and local children.

## Methods

### Study design and participants

A cross-sectional survey was conducted among children aged under 12 months in mainland China in 2018, which was described in detail below. Their caregivers were interviewed about breastfeeding practices and potential determinants face-to-face.

Multi-stage stratified cluster random sampling was used in the study. All counties/districts (2854 in total) in 31 provinces/autonomous regions/municipalities in China were categorized into four strata: metropolis, medium and small cities, general rural areas and poor rural areas. Based on the population within 1 year of the National Notifiable Disease Report System in 2014, Probability Proportional to Size (PPS) sampling was used to select counties from each stratum. After considering geographic distribution, 12 counties were chosen in the study (four metropolis, four medium and small cities, two general rural areas and two poor rural areas). In each selected county, four townships or communities were PPS sampled and additional sampling was conducted from adjacent county if the number of communities was less than four in some medium and small cities. Finally, in each selected township, 70 children from each month- age group were randomly selected according to list of children in the Expanded Program on Immunization. In metropolis and medium and small cities, household registered children and migrant children whose mothers migrated from other counties for more than 1 month were sampled in the same proportion. The total sample size was 9760 for children under 1 year, of which 6995 was for children from metropolis and medium and small cities. Sample size calculation was based on the EBF rate according to the Chinese Residents’ Nutrition and Health Survey in 2013 and complex sampling design was considered.

### Measurements

Uniformly trained investigators collected data by face-to-face interviews, using smartphone/pad-based questionnaires to record response. All the questionnaires were thoroughly checked up for omissions or errors and corrected during the interview. Quality control staffs from the provincial CDCs checked these questionnaires for another time.

Maternal rural-to-urban migrant status was the primary variable in this study. Considering that rural-to-urban migrants were mainly found in cities, we excluded participants from the rural areas in this paper. In the baseline survey, mothers were asked about their current place of residence and time of residence. The place of residence was classified into six categories, including non-household registration residence at province level, non-household registration residence at city level, non-household registration residence at county level, non-household registration residence at village level, household registration residence and others. The duration of residence was classified into three categories, including less than 1 month, 1 to 5 months and more than half a year. In this study, rural-to-urban migrant was defined as migrating from the household registration residence at county level or above for more than 1 month, according to the definition from National Health and Family Planning Commission [17]. Those who migrated for less than a month were excluded in our study due to their unstable residence status.

The outcome variables in this study were ever breastfeeding, exclusive breastfeeding (EBF) under 6 months, predominant breastfeeding under 6 months and age-appropriate breastfeeding. These breastfeeding practices were assessed based on the World Health Organization indicators for assessing infant and young child feeding practices [18]. The calculation of EBF and predominant breastfeeding was conducted among infants aged under 6 months. Exclusive breastfeeding was defined as breast milk was the only food and liquid consumed with the exception of oral rehydration salt, drops and syrups (vitamins, minerals and medicines), using a 24-h recall questionnaire. Predominant breastfeeding was defined as proportion of infants aged under 6 months who received both breast milk and other fluids but not milk or solid foods during the previous day. For Ever breastfeeding and age-appropriate breastfeeding prevalence, the calculation was conducted among children aged from 0 to 12 months. Ever breastfeeding was defined as proportion of children born in the last 12 months who were ever breastfed. Age-appropriate breastfeeding prevalence was calculated using the sum of infants aged under 6 months who were exclusively breastfed during the previous day and children aged 6–12 months who received breast milk with solid, semi-solid or soft foods during the previous day divided by the total number of children aged 0–12 months.

Sociodemographic characteristics were grouped into two categories: mothers and infants. Mothers’ sociodemographic characteristics included maternal age, maternal ethnicity, maternal body-mass index (BMI), maternal education level, maternal employment and maternal residence. Those of infants were infant sex, infant birthweight, delivery method and preterm birth. Health information included maternal illness during pregnancy and infant’s illness within 2 weeks after birth. According to the mothers’ self-reported questionnaire, they were considered not ill during pregnancy unless they’ve had gestational diabetes mellitus or hypertensive disorder complication pregnancy or postpartum hemorrhage. Infants was considered ill within 2 weeks after birth if they had one of: neonatal hospitalization, neonatal hypoglycemia, neonatal jaundice, diarrhea within 2 weeks after birth and respiratory diseases within 2 weeks after birth. Otherwise, they were considered healthy within 2 weeks after birth. Supportive information were those covariates related to knowledge, experience and support to breastfeeding, including maternal breastfeeding history (whether they had ever breastfed a child), maternal knowledge about benefits of breastfeeding, parents supported breastfeeding, parents-in-law supported breastfeeding, husband supported breastfeeding and best friend supported breastfeeding. Maternal knowledge about benefits of breastfeeding was measured using a 11-items multiple-choice question and categorized into two groups: above upper quantile (scored better than 75% mothers) and below upper quantile. Mothers were asked about their families and best friend’s attitude about breast milk and infant formula milk. They were considered supporting breastfeeding only if they thought breast milk was much better than formula milk. Otherwise they were not considered supporting breastfeeding.

### Data analysis

Pairwise deletion was used to handle missing data in statistical descriptions or univariate analysis. In multivariable analyses, we used listwise deletion for variables with < 10% missing data, and assigned a category “unknown” to the missing value of categorial variables with ≥10% missing data and did not excluded these cases.

Descriptive analysis was used to illustrate the basic sociodemographic characteristics in different maternal rural-to-urban migrant groups. The independent t-test and χ^2^ test were used to compare the distributions of sociodemographic characteristics according to different migrant status as appropriate.

The prevalence of breastfeeding practices was compared according to maternal rural-to-urban migrant status and Pearson χ^2^ test was used to test the significance. The associations between maternal rural-to-urban migrant status and breastfeeding outcomes were analyzed using logistic regression model fitted: (1) unadjusted; (2) adjusted for mother-infant sociodemographic characteristics; (3) additionally adjusted for mother-infant health information; (4) further adjusted for supportive information. To further understand the relationship between maternal rural-to-urban migrant status and their breastfeeding practices, we stratified them into different groups using their education level, place of residence and ethnicity, respectively and logistic regression models mentioned above were conducted according to these stratifications. In this step, to simplify the stratification, maternal education level was further categorized into three groups: “high” including college and above; “mid” including senior high school; “low” including junior high school and below, which were compulsory education in China. A stepwise backward elimination procedure was carried out in the regression model and the odds ratios were presented with 95% confidence interval.

All of the analyses were done with R 3.6.0 and SPSS 24.0. Two-sided *p* values of less than 0.05 were deemed to be statistically significant.

## Results

Table 1 shows the sociodemographic characteristics of mothers and infants by maternal rural-to-urban migrant status. Of all 6995 participants from metropolis and medium sized or small cities, 6896 (98.6%) mothers were included in our study (49 were excluded for missing data and 50 for unstable residence status). 4381 (65.5%) of them were local population and 2565 (34.5%) were rural-to-urban migrants.

**Table 1:** Sociodemographic characteristics of mothers and infants in different maternal rural-to-urban migrant status

	Maternal rural-to-urban migrant status		*p*-value
	Local (*n* = 4381)	Migrant (*n* = 2515)	
Maternal age, *n* (%) ^a^			< 0.001
< 25	577 (13.20)	433 (17.28)	
25-	1562 (35.74)	971 (38.75)	
30-	1265 (28.94)	741 (29.57)	
35-	778 (17.80)	297 (11.85)	
40–53	189 (4.32)	64 (2.55)	
Maternal BMI, *n* (%) ^b^			0.033
Underweight (< 18.5 kg/m^2^)	579 (13.98)	351 (15.07)	
Normal weight (18.5–23.9 kg/m^2^)	2671 (64.49)	1544 (66.29)	
Overweight (24.0–27.9 kg/m^2^)	651 (15.72)	326 (14.00)	
Obesity (≥ 28.0 kg/m^2^)	241 (5.82)	108 (4.64)	
Infant birthweight, *n* (%) ^c^			0.288
< 2500 g	164 (3.79)	81 (3.26)	
2500–3999 g	3775 (87.28)	2202 (88.54)	
≥ 4000 g	386 (8.92)	204 (8.20)	
Infant sex, *n* (%)			0.627
Male	2224 (50.76)	1292 (49.24)	
Female	2157 (51.37)	1223 (48.63)	
Maternal education level, *n* (%) ^d^			0.607
Primary school and below	264 (6.03)	158 (6.29)	
Junior high school	1071 (24.48)	638 (25.40)	
Senior high school	859 (19.63)	464 (18.47)	
College and above	2181 (49.85)	1252 (49.84)	
Occupation, *n* (%) ^e^			< 0.001
Unemployment	1562 (35.69)	937 (37.35)	
Informal employment	1410 (32.21)	932 (37.15)	
Formal employment	1405 (32.10)	640 (25.51)	
Ethnicity, *n* (%) ^f^			< 0.001
Han	3656 (83.57)	1996 (79.59)	
Others	719 (16.43)	512 (20.41)	
Place of residence, *n* (%)			< 0.001
Metropolis	1910 (43.60)	1639 (65.17)	
Medium sized/small cities	2471 (56.40)	876 (34.83)	
Delivery method, *n* (%) ^g^			< 0.001
Vaginal	2618 (59.92)	1615 (64.29)	
Caesarean section	1751 (40.08)	897 (35.71)	
Preterm birth, *n* (%) ^h^			0.140
No	4095 (95.19)	2377 (95.96)	
Yes	207 (4.81)	100 (4.04)	

^a^19 cases with missing data of maternal age. ^b^425 cases with missing data of maternal BMI. ^c^84 cases with missing data of infant birthweight. ^d^9 cases with missing data of maternal education level. ^e^10 cases with missing data of maternal occupation. ^f^13 cases with missing data of maternal ethnicity. ^g^15 cases with missing data of delivery method. ^h^117 cases with missing data of preterm birth

The mean maternal age of rural-to-urban migrants was significantly smaller than that of local population (29.05 vs. 30.07, *p* <  0.001) and so was the maternal BMI (21.73 for migrants vs. 21.95 for local people, *p* = 0.033).

The distributions of infant birthweight, infant sex, maternal education and preterm birth were similar between local people and migrants.

As for occupation, after conducting a post-hoc test, we found migrant mothers were less likely to get formal employment. Local mothers were more likely to be Han ethnic, living in medium sized or small cities and having vaginal deliveries.

As shown in Table 2, the overall prevalence of ever breastfeeding, EBF, predominant breastfeeding and age-appropriate breastfeeding were 97.51% (6724/6896), 29.84% (1049/3516), 59.89% (2099/3505) and 45.07% (3108/6896), respectively. Compared to local people, the prevalence of breastfeeding practices of rural-to-urban migrants were slightly but not significantly lower. However, significant differences were found in ever breastfeeding and EBF between the two groups after adjusting for mother-infant sociodemographic characteristics and mother-infant health information. However, only EBF was still significantly less among migrants when further adjusted for mothers who received supportive information (OR 0.81, 95% CI 0.68, 0.95). No significant differences were found in other two breastfeeding practices.

**Table 2:** Adjusted ORs for breastfeeding practices according to maternal rural-to-urban migrant status

Outcome	*N* (%)	Model A	Model B	Model C
		AOR (95% CI)	AOR (95% CI)	AOR (95% CI)
Ever breastfeeding				
Local	4276 (97.60)	1.00 (reference)	1.00 (reference)	1.00 (reference)
Migrant	2448 (97.34)	0.70 (0.50, 0.97)	0.70 (0.50, 0.97)	0.70 (0.50, 1.00)
EBF^a^				
Local	688 (30.32)	1.00 (reference)	1.00 (reference)	1.00 (reference)
Migrant	361 (28.95)	0.83 (0.70, 0.98)	0.79 (0.67, 0.94)	0.81 (0.68, 0.95)
Predominant breastfeeding^a^				
Local	1359 (60.03)	1.00 (reference)	1.00 (reference)	1.00 (reference)
Migrant	740 (59.63)	0.93 (0.81, 1.08)	0.92 (0.80, 1.07)	0.93 (0.80, 1.08)
Age-appropriate breastfeeding				
Local	1990 (45.42)	1.00 (reference)	1.00 (reference)	1.00 (reference)
Migrant	1138 (45.25)	0.97 (0.87, 1.07)	0.96 (0.86, 1.06)	0.97 (0.87, 1.08)

*AOR* Adjusted odds ratio

Model A: odds ratio was adjusted for mother-infant sociodemographic characteristics (maternal age, maternal education levels, maternal occupation, maternal ethnicity, maternal residence, infant sex, infant birthweight, delivery method and preterm birth)

Model B: odds ratio was additionally adjusted for mother-infant health information (maternal illness during pregnancy, infant illness within 2 weeks after birth)

Model C: besides the covariates adjusted in model B, supportive information (maternal history of EBF, maternal knowledge about EBF, grandparents support EBF, grandparents in law support EBF, fathers support EBF and mothers’ best friends support EBF) were also adjusted in this model

^a^prevalence and odds ratios for EBF and predominant breastfeeding were calculated among infants aged under 6 months

Table 3 shows the relationships between rural-to-urban migrant status and EBF stratified by maternal education level, place of residence and maternal ethnicity, respectively. In the stratification of maternal education level, the overall prevalence of EBF was not significantly different between rural-to-urban migrants and local people in both groups. However, after adjusting for other variables, rural-to-urban migrants showed lower EBF rate than local people merely in the group with high education level (OR 0.79, 95% CI 0.63, 0.98). Referring to the stratification of place of residence, significantly lower EBF rate in rural-to-urban migrants was only noticed among those living in metropolis (OR 0.81, 95% CI 0.66, 0.99). Stratified by maternal ethnicity, rural-to-urban migrant children of minority mothers were considerably less exclusively breastfed than local children of minority mothers (OR 0.60, 95% CI 0.41, 0.87).

**Table 3:** Relations between maternal rural-to-urban migrant status and EBF stratified by different sociodemographic characteristics

	*N* (%)		*p*-value	Adjusted OR (95% CI) ^a^	
	Local	Migrant		Local	Migrant
Education ^b^					
High	406 (35.37)	224 (34.51)	0.716	1.00 (reference)	0.79 (0.63, 0.98)
Mid	109 (25.17)	53 (23.87)	0.715	1.00 (reference)	0.75 (0.50, 1.14)
Low	172 (25.11)	84 (22.34)	0.314	1.00 (reference)	0.86 (0.63, 1.16)
Residence					
Metropolis	378 (37.95)	276 (33.50)	0.049	1.00 (reference)	0.81 (0.66, 0.99)
Medium sized/small cites	310 (24.35)	85 (20.09)	0.073	1.00 (reference)	0.77 (0.58, 1.02)
Ethnicity					
Han	565 (29.72)	302 (30.57)	0.999	1.00 (reference)	0.86 (0.71, 1.03)
Minority	123 (33.70)	59 (22.87)	0.010	1.00 (reference)	0.60 (0.41, 0.87)

^a^The adjusted variables are the same variables adjusted in the Model C, Table 2

^b^1 case with missing data of maternal education level

## Discussion

This is the first study to describe prevalence of breastfeeding practices among rural-to-urban migrants and examine the association between them. Based on a large, population-based sampling survey, the findings in this article are highly reliable and meaningful. In the present study, the prevalence of breastfeeding practices was not optimal to meet the goal by 2020. The rural-to-urban migration was only inversely related to EBF among those with high education level, living in metropolis and being minority, after adjusting for other covariates.

### Prevalence of breastfeeding practices

The overall prevalence of breastfeeding practices found in this study is too low to meet the goal about 50% EBF rate in 2020. Recent study showed that only 37% of children younger than 6 months of age were exclusively breastfed in low-income and middle-income countries [2], higher than what we found in this study. Chinese used to have a good tradition of breastfeeding and more than 80% mothers breastfed their children [19]. But since 1970s, the prevalence of breastfeeding has dropped dramatically [3, 20]. There is urgent need for promotion methods, like personal sessions, family education and social support, to increase breastfeeding rate in China, especially EBF rate [21–23].

### Association between breastfeeding practices and rural-to-urban migration

In the present study, we found that prevalence of the four breastfeeding practices (ever breastfeeding, EBF, predominant breastfeeding and age-appropriate breastfeeding) in rural-to-urban migrants was slightly, but not significantly lower than that of local population. Rural-to-urban migrants are more vulnerable to health-related issues because most health services and policies are registration-related in China, making those migrants inconvenient to have access to them. However, breastfeeding is more complicated than those traditional health issues. It also takes consideration of self-efficacy and social environment [23–25]. Some studies concluded that maternal education was a strong indicator for breastfeeding practices [26]. We found no difference in maternal education between the two groups of participants, which could reduce the difference in breastfeeding practices between them. Baby-friendly hospital practices may also help the establishment of breastfeeding [27], particularly giving only breast milk in the hospital [28]. As the new Medicare Reform involved more registration-inconsistent people, rural-to-urban migrants can have similar, even the same access to hospital services like local citizens, resulting in similar prevalence of breastfeeding practices in the two groups.

After adjusting for other confounding variables, rural-to-urban migrant status was solely, significantly and robustly associated with exclusive breastfeeding. This indicates the association between rural-to-urban migrant status and EBF may be modified by other variates. In another study conducted in 2013 in China, they examined the association between maternal migrant status and EBF and found it not significant [4]. However, the definition of maternal migrant status was not described in the study and no further multivariate analysis was conducted between them. We found no other study discussing the association between rural-to-urban migrant status and breastfeeding practices in China.

According to previous studies, maternal education level, place of residence and maternal ethnicity were three main factors that associated with breastfeeding prevalence [29]. Stratified by those three factors, we noticed some special, vulnerable groups which should be paid more attention to about exclusive breastfeeding. Migrants with high education level gave less exclusive breastfeeding to their children than local mothers with the same diploma. However, such difference was not found within those with low education level. The association between maternal education level and EBF remains inconsistent. According to the systematic review of Boccolini et al., the low education level was associated with the interruption of EBF in Brazil [29]. However, another systematic review of Zhao J et al. found that in the Chinese culture and employment environment, mother with higher education level were less likely to breastfeed their babies compared to those who were less educated [26]. Among higher educated mothers in our study, rural-to-urban migrant status became a risk factor for EBF and we presumed that it was caused by their working status and living places. Migrants are more likely to rent a house, rather than buying one, without enough space for breastfeeding, especially in suburb areas. Higher educated migrant mothers may have better jobs, equivalent to their diploma, in central areas and hire babysitters to looking after their children. As migrants, they may face more pressure than local citizens and have to work hard to avoid being fired and earn enough money for rental and baby caring. Thus, they may have difficulties balancing work and child care and reducing their time with children [30, 31]. The long distance between their rent house and working place also reduced their time for breastfeeding. Migrants with lower education level may just find jobs near their living place for convenience and most of them may be informal employed, which means they have less traffic time and face less pressure than higher educated ones [32]. The low EBF rate of lower educated participants itself may also lighten the influence of rural-to-urban migration on exclusive breastfeeding.

Place of residence is another factor that affects the association between rural-to-urban migration and exclusive breastfeeding. Overall, living in metropolis rather than medium sized or small cities can promote exclusive breastfeeding. Super cities, like capitals, can provide more health-related services, prenatal lessons and peer education to highlight the importance of exclusive breastfeeding [33]. In the subgroup of participants living in metropolis, rural-to-urban migrant children are less likely to be exclusively breastfed than local ones. The expense of living in metropolis is much higher for migrants and they are more common to have mental health problems than natives [34]. When local citizens are surrounded by plenty resources of postpartum and neonatal caring, the migrants may be facing heavy working load or traffic jam, resulting in less time for breastfeeding. High prevalence of postpartum mental health problems in migrants can also deter the execution of exclusive breastfeeding [35]. Duration of residence can also affect breastfeeding practices. A study conducted in Hong Kong revealed that breastfeeding duration was progressively shorter when the immigrant time increased [14]. However, the difference of EBF rate between rural-to-urban migrants and local people living in medium sized or small cities is slight but not significant. The scale of the cities is not as big as metropolis and the traffic congestion is not so severe, so these migrants waste less time in commuting and have enough time for breastfeeding. Also, migrants living in medium sized or small cities face less stress of living and working than those in big cities and they are less vulnerable to postpartum mental health problems.

The lower prevalence of EBF in rural-to-urban migrants was solely apparent in ethnic minorities (other 55 ethnicities except for Han). This result is consistent with previous findings. Fenglian Xu et al. conducted a survey in Xinjiang, China 2004 and concluded that EBF rate in the Han was significantly lower than “other minority” (excluding Uygur) [36]. There was only 1 Uygur participant in our study, making our “minority” similar to “other minority” in Xu’s article. Another study in China 2014 also found ethnic Han was associated with decreased likelihood of exclusive breastfeeding [37]. Acculturation to the culture of not favoring breastfeeding can reduce the prevalence of exclusive breastfeeding [38]. We postulated that migrants were likely to abandon their traditional breastfeeding practice and adopt Han’s disfavor of breastfeeding [37, 39]. Local minority citizens are more likely to live in ethnic communities and maintain their traditional breastfeeding habits [40]. However, it might be hard for minority migrants to fit into such ethnic communities, resulting in loss of their traditional breastfeeding practices.

There are still some limitations in our study. First, we didn’t consider the original registration place for migrant population specifically. Where they are from may largely affect their employment status, then make difference to their practice of breastfeeding. Second, causal effect between rural-to-urban migrant status and EBF was difficult to examine because of the nature of cross-sectional study. Further perspective study is needed to clarify the causal relationship. Third, the prevalence of breastfeeding practices was calculated based on a “24-hour recall method”, which could overestimate the prevalence and cause recall bias.

## Conclusions

A large proportion of infants were not exclusively breastfed in both local and rural-to-urban migrant population. The prevalence of breastfeeding practices was lower than the WHO recommendation and the target of Chinese National Nutrition Plan (2017–2030). This study has indicated rural-to-urban migrant status is a risk factor for mothers to practice exclusive breastfeeding. The association between migrant status and prevalence of EBF varied across maternal education level, place of residence and maternal ethnicity. Besides common strategies, special approaches should be provided for those vulnerable groups.

## Data Availability

After the article is published, data without hospital names and identifiers (but not the study protocol, statistical analysis plan, or informed consent form) will be available to researchers who submit a proposal to the corresponding authors with a signed data access agreement. The corresponding authors have the right to decide whether to share the data or not based on the research objectives and plan provided.

## Abbreviations

BMI: Body mass index
EBF: Exclusive breastfeeding
WHO: World Health Organization
